#  A quality improvement plan for hypertension control: the INCOTECA Project (INterventions for COntrol of hyperTEnsion in CAtalonia)

**DOI:** 10.1186/1471-2458-9-89

**Published:** 2009-03-25

**Authors:** Roser Vallès-Fernandez, Magdalena Rosell-Murphy, Olga Correcher-Aventin, Lucas Mengual-Martínez, Núria Aznar-Martínez, Gemma Prieto-De Lamo, Alícia Franzi-Sisó, Jordi Puig-Manresa, Josep Ma Bonet-Simó, the INCOTECA research group

**Affiliations:** 1 CAP Fontetes, Primary Care Service (SAP) Cerdanyola-Ripollet, Catalan Institute of Health (ICS), Pl Velázquez s/n 08291 Cerdanyola del Vallès, Barcelona, Spain; 2 Primary Care Research Institute (IDIAP Jordi Gol), c/Gran Via de les Corts Catalanes, 587 àtic, 08007 Barcelona, Spain; 3 Primary Care Team (EAP) Badia del Vallès, Catalan Institute of Health (ICS), c/Bética s/n, 08214 Badia del Vallès, Barcelona, Spain; 4 Primary Care Team (EAP) Serraparera, Catalan Institute of Health (ICS), c/Diagonal s/n 08290 Cerdanyola del Vallès, Barcelona, Spain; 5 Research Support Unit (USR) Centre, Catalan Institute of Health (ICS), C/Torrebonica s/n 08277 Terrassa, Barcelona, Spain; 6 Primary Care Service (SAP) Sabadell-Rubí-St, Cugat-Terrassa, Catalan Institute of Health (ICS), c/Ctra Barcelona 473 08204 Sabadell, Barcelona, Spain

## Abstract

**Background:**

Different studies have shown insufficient blood pressure (BP) control in hypertensive patients. Multiple factors influence hypertension management, and the quality of primary care is one of them. We decided therefore to evaluate the effectiveness of a quality improvement plan directed at professionals of Primary Health Care Teams (PHCT) with the aim to achieve a better control of hypertension. The hypothesis of the study is that the implementation of a quality improvement plan will improve the control of hypertension. The primary aim of this study will be to evaluate the effectiveness of this plan.

**Methods and design:**

*Design*: multicentric study quasi-experimental before – after with control group. The non-randomised allocation of the intervention will be done at PHCT level.

*Setting*: 18 PHCT in the Barcelona province (Spain).

*Sample*: all patients with a diagnosis of hypertension (population based study). Exclusion criteria: patients with a diagnosis of hypertension made later than 01/01/2006 and patients younger than 18 years.

*Intervention*: a quality improvement plan, which targets primary health care professionals and includes educational sessions, feedback to health professionals, audit and implementation of recommended clinical practice guidelines for the management of hypertensive patients.

*Measurements*: age, sex, associated co-morbidity (diabetes mellitus type I and II, heart failure and renal failure). The following variables will be recorded: BP measurement, cardiovascular risk and antihypertensive drugs used. Results will be measured before the start of the intervention and twelve months after the start of the study.

*Dependent variable*: prevalence of hypertensive patients with poor BP control.

*Analysis*: Chi-square test and Student's t-test will be used to measure the association between independent qualitative and quantitative variables, respectively. Non-parametric tests will be used for the analysis of non-normally distributed variables. Significance level (α) will be set at < 0.05. Outcomes will be analysed on an intention-to-treat basis.

**Discussion:**

The implementation of a quality improvement plan might benefit the coordination of different professionals of PHCTs and may also improve blood pressure control.

**Trial Registration:**

This protocol has been registered at clinicaltrials.gov with the ID number MS: 1998275938244441.

## Background

Hypertension is one of the most important risk factors of cardiovascular morbidity and mortality and one of the main reasons for seeking medical attention in primary care, especially in the elderly population [1-5]. The prevalence of hypertension in Spain ranges from 20 to 47% in the population older than 20 years and up to 65% in the population above 60 years of age [2,4].

Since hypertension is a powerful risk factor of survival from cardiovascular disease, its control is highly important. In Spain, one out of two deaths of cardiovascular origin that occur yearly in patients above 50 years of age can be attributed to high blood pressure, and in 90% of cases to hypertension [6].

Patients with uncontrolled hypertension usually present with other cardiovascular risk factors, more target organ damage, and an increased number of cardiovascular diseases associated with their condition [7,8].

A number of studies carried out both in Europe and in the United States have shown that blood pressure (BP) control in hypertensive patients is suboptimal [2,9-15]. In Spain a study revealed that BP was properly controlled only in 33.5% of the patients older than 65 years [2]. In a separate study that took place in Catalonia (specifically in the Àmbit Centre of the Catalan Institute of Health (ICS) where we aim to develop this project), the proportions of properly controlled hypertensive patients above 15 years of age with and without diabetes were 23.9% and 44.3%, respectively [16]. Although higher than those reported in other studies [7,10,17], these proportions remain sub-optimal. However, a recent study of the hypertensive population in Spain indicates an improving trend in the control of this condition [10].

Different factors have been associated with an inadequate control of hypertension [2,9-15,18-25]:

1. Patient-related factors: therapeutic adhesion, age, lifestyle (alcoholism, sedentarism), body-mass index, number of visits to the doctor during the past year and the understanding of their condition, among others.

2. Treatment-related factors: it is becoming increasingly obvious that for many patients the use of monotherapy is not sufficient to control BP; other concomitant treatments the patient might be receiving can interfere with antihypertensive drugs; the specific antihypertensive drug chosen taking into account the degree of hypertension and the associated conditions.

3. Factors related to the clinical management: the technique to measure BP and the time of day this measurement is done (morning, afternoon, before or after medication), as well as the clinical management of uncontrolled hypertensive patients. It is equally essential to follow clinical guidelines to avoid clinical inertia, such as when there is no treatment intensification in uncontrolled hypertension even when there is good therapeutic compliance.

4. Factors related to the equipment used to measure the BP, such as the type of monitor (aneroid, automatic, mercury), its precision, validation and calibration.

Care delivered by primary health centres is one of the factors that can influence the control of hypertensive patients [26]. The implementation of some measures that affect both patients and health professionals are necessary if a better control of hypertension is intended. Therefore, an improvement is required in the following areas: information and knowledge about the techniques to measure BP; the follow-up of patients; analysis of patient compliance and of the implementation of treatment guidelines, where continued education and patient positive feed-back are considered crucial [12,27].

Based on the published medical literature, a research project that considers an integral, multicentric and multidisciplinary intervention at primary health care level with an impact on daily practices of both clinicians and nurses, may improve the control of hypertension. Previous studies describing the implementation of interventions based on methods to improve the quality of health care have been shown to have a positive impact on health [28]. Subsequently, we believe it is important to assess the effectiveness of a programme to better the quality of health care, addressed to primary health care professionals with the aim to optimise the control of BP in hypertensive patients.

### Hypothesis

The hypothesis that underlies this project states that the implementation of a plan for quality improvement at PHC level addressed to primary health care professionals will ameliorate the management and control of hypertensive patients.

### Aims

#### Primary aim

to assess the effectiveness of a programme directed to health professionals which intends to improve the quality of health care to optimise the control of BP in hypertensive patients.

#### Secondary aims

to assess the impact on uncontrolled hypertension of the presence or absence of co-morbidities and the number of antihypertensive drugs prescribed.

## Methods and design

### Study design

**Figure 1:**
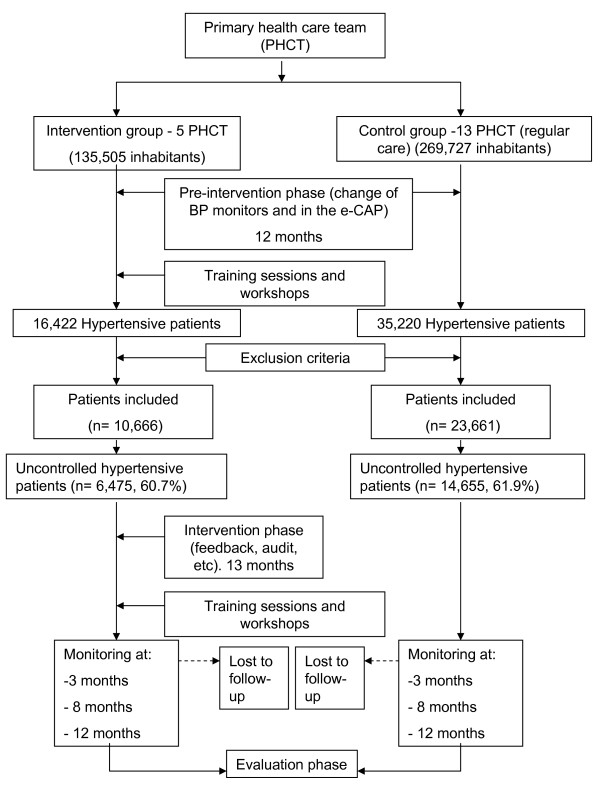
**General Outline of the Study**.

The study will be a multicentric non-randomised controlled quasi-experimental pre-post intervention study to assess the effectiveness of a quality improvement plan at PHC level (see figure 1). The non-randomised allocation of the subjects will be decided at the PHCTs.

### Setting

Intervention group: 5 PHCTs of the Primary Health Care area of Cerdanyola-Ripollet (ICS) with 135,505 inhabitants in the catchment area at the onset of the study.

Control group: 13 PHCTs of the Primary Health Care area of Sabadell (ICS) with 269,727 inhabitants in the catchment area at the onset of the study.

Both groups are comparable in terms of population characteristics and socio-economical level. Availability of computerised clinical records in the PHCTs before January 2005 will be an inclusion criterion.

Both groups of health professionals (control and intervention) have been informed about the nature of the study, and have given a verbal consent and agreement to participate in the study.

### Study population

This is a population-based study that will include all patients diagnosed with hypertension.

Exclusion criteria:

1. Patients diagnosed with hypertension after January 1^st^, 2006.

2. Patients under 18 years of age.

3. Patients with a diastolic blood pressure (DBP) higher than systolic blood pressure (SBP) as recorded in the clinical records.

### Definition of uncontrolled hypertension

1. SBP ≥ 140 mmHg and/or DBP ≥ 90 mmHg. In patients with diabetes, heart failure or renal failure, SBP ≥ 130 mmHg and/or DBP ≥ 85 mmHg. In all cases the values will be taken from the measurements recorded at the patient's last visit to the primary health centre.

2. Absence of any recorded BP measurement in the computerised clinical record within the past 12 months.

### Measurements

In addition to age and sex, and to ensure the comparability of the groups studied, the following variables will be collected: co-morbidities associated with hypertension (presence of diabetes mellitus type I or II, heart failure or renal failure), cardiovascular risk assessment according to the Framingham tables [29], the presence of cardiovascular events, and duration of hypertension. The following dependent and independent variables will be collected from the control and intervention groups: BP measurement, register of cardiovascular risk in PHC prevention, presence of a cardiovascular event, participation in health care education, number of BP readings within the past 12 months, and treatments received. Therapeutic compliance will be evaluated by the Morisky-Green test [30], the self-report compliance Haynes-Sackett test [31], and searching the computer databases for pharmaceutical prescription.

### Dependent or outcome variable

In agreement with the main aim of this study, the dependent or main outcome variable will be good control of hypertension as a dichotomous variable (yes/no) based on SBP and DBP records over the last 12 months, or alternatively, the last values of SBP and DBP readings at the time the data is analysed.

### Independent variables and effect modifiers

The association of the following independent variables with the main dependent variable will be assessed: age, sex, treatment with antihypertensive drugs (yes/no), number of antihypertensive drugs, co-morbidities (yes/no), assessment of therapeutic compliance (yes/no), health education (yes/no), cardiovascular risk assessment according to the Framingham tables, duration of hypertension, and number of SBP and DBP readings within the past 12 months.

### Intervention

**Figure 2:**
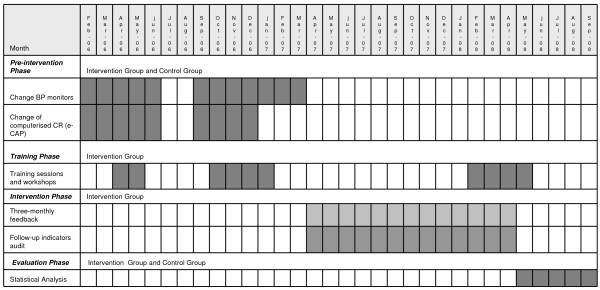
**Timetable**.

The study intervention will be the implementation of a plan of quality improvement addressed to health professionals in PHCTs. The quality improvement plan consists of the following phases:

1. ***Pre-intervention phase ***(12 months): in order to ensure that hypertension is diagnosed under the same conditions in both the intervention and the control groups, the following actions will take place:

a) Non-validated blood pressure monitors will be removed from the PHCT examination rooms. This will allow us to standardise the equipment used to measure BP in both the control and the intervention groups. The model used in this study will be a digital Omron-6 blood pressure monitor, which is the model approved and recommended in the Catalan Institute of Health (ICS) hypertension clinical guidelines.

b) The software used to store computerised clinical records will be modified to allow health professionals to adequately enter specific data related to hypertensive patients (such as the control arm or the results from the Morisky-Green therapeutic adhesion evaluation test). This software is used by all health professionals working for the ICS, thus it will be used by health care professionals in the PHCTs of both the intervention and the control groups.

c) Make the Catalan Institute of Health (ICS) guidelines on hypertension accessible to health professionals. These are the reference clinical guidelines for all health professionals that will participate in this study.

2. ***Second phase ***(training, 10 months): a programme has been designed to train health professionals of PHCTs (doctors and nurses) on how to diagnose hypertension correctly, the technique required to adequately measure BP, and how to enter patient data in computerised clinical records. This programme will be delivered through training seminars and workshops. Training material, such as posters and leaflets with decision-making algorithms as recommended by the ICS clinical guidelines, will be made available to participants. These workshops will be carried out by trained professionals (doctors and nurses) from the same PHCTs that participate in the study. There will be a workshop for each of the participating PHCTs on each of the topics described above. By the end of the programme a total of 8 workshops will have taken place at each of the participating PHCTs.

3. ***Third phase ***(intervention, 13 months): the interventions carried out during this phase will aim at identifying uncontrolled hypertensive patients and act on them to improve the control of their condition. This will be accomplished by:

a) Every 6 months health professionals will receive feedback on uncontrolled hypertensive patients with individualised information about the degree of control and antihypertensive treatment. These data will be retrieved from the computerised clinical records. In collaboration with the administrative staff a specific track to get these patients an appointment to see a doctor or nurse will be devised. Patients with uncontrolled hypertension that are not on any antihypertensive drug will get an appointment to see the doctor and to evaluate if antihypertensive treatment is indicated. Patients receiving antihypertensive drugs will get an appointment with the nurse to assess adhesion to treatment and compliance with the recommended hygienic and dietetic measures. Improvement strategies will be devised if patients fail to comply with these measures. Examples of such strategies would be the implementation of drug dosing methods that remind patients to take the medication, the improvement of communication with patients to increase the understanding of the treatment they receive, and detection of adverse effects that may decrease therapeutic compliance. Patients with good therapeutic compliance will be referred to the doctor who will assess the pharmacological treatment (drug associations and interactions) according to the current clinical guidelines.

b) An audit will be undertaken to assess the implementation of the improvement plan and the adhesion to the recommended clinical guidelines, i. e. retrieval every six months of patient information from computerised clinical records to generate indicators of study protocol adhesion and dissemination amongst health professionals.

c) The dissemination of the data to health professionals and preliminary analysis will be coordinated by the research team.

d) Support Team: each PHCT will have a doctor and a nurse that will provide support to the rest of professionals of the PHCT on any aspect of the programme implementation. This team will also facilitate the communication and coordination with the research team.

4. ***Fourth phase ***(evaluation of the implementation of a quality improvement plan at 4 months): after the conclusion of the third phase a pre-post treatment analysis will be carried out to evaluate the effectiveness of the quality plan. The results from this analysis will be compared to those for the control group during the same period. Figure 2 shows the time line for the implementation of the different phases.

### Control group

Health professionals for the control group use the same software to enter patient data in computerised clinical records and follow the same clinical guidelines for the management of hypertensive patients as the intervention group. The follow up of hypertensive patients will be done as usual in the PHC centres, though health professionals will not receive any feedback on the adequacy of the control of their patients.

### Analytical plan

Data will be reported according to the CONSORT statement for reporting of cluster randomised trials [32] and comparisons will be based on intention-to-treat.

A baseline description and comparison of the two groups will be carried out in relation to the main variables studied. Student's t-test and Mann-Whitney U-test (according to the frequency distribution of the variables studied) will be used to compare the means of the two groups. ANOVA will be used to compare means when more than two categories exist. Chi-square test or chi-square for trend will be used to compare proportions for categorical variables. Logistic regression analysis will be carried out to model the association of the dependent variable (uncontrolled hypertension) with the independent variables selected from the univariate comparisons. All analyses will be adjusted for potential confounders and the relevant clinical variables. The analysis will be done with SPSS version 15 for Windows and performed at the 95% confidence level (two-sided).

The quality improvement plan will be considered effective if at least an increase in 10% is observed in the proportion of hypertensive patients adequately controlled compared with the baseline proportion observed in the intervention group.

### Ethical considerations

The study will be performed following good clinical practice (GCP) guidelines and complying with the Declaration of Helsinki principles. The study protocol has received institutional review board approval (Ethical Clinical Committee at the IDIAP Jordi Gol).

## Discussion

Previous studies have shown that the implementation of multifactorial programmes for the management of different conditions have a positive impact on health [28,33]. The aim of the INCOTECA Project is to create a programme to manage hypertension and to set up a working routine within PHCTs to improve the health of hypertensive patients and to evaluate subsequently its effectiveness.

A population-based quality improvement plan to identify and resolve problems that not only targets daily practices of doctors and nurses but also teams up with administrative staff at health centres and addresses the main factors associated with a poor control of hypertensive patients is likely to have a positive impact in the control of this condition. Moreover, it is expected that it will eventually alleviate the health burden caused by these patients.

The quality improvement plan is a multidisciplinary programme that intends to involve the participation of doctors and nurses as well as pharmacists and administrative staff. This plan includes a number of measures that should affect various aspects of patient management in the PHCTs, such as structural measures (optimisation of the equipment for patient follow-up), organisational measures (specific plan to schedule appointments for uncontrolled hypertensive patients), educational measures (a training plan for health care staff) and measures that affect clinical practice (assessment of therapeutic compliance and health education for patients with poorly controlled hypertension).

The main limiting factors to assess the effectiveness of this plan are:

- *Variability in the implementation and data entry in computerised clinical records*: the use of standardised software to enter patient data in computerised clinical records is not occurring at the same pace in the different PCHTs that will participate in this study. In order to minimise this problem, it will be required as part of the inclusion criteria to participate in this study that the PHCTs have been using computerised clinical records since January 2005 to give health professionals in these teams sufficient time to familiarise themselves with this tool and to have accrued sufficient data to carry out the improvement plan.

- *Variability in the technique to measure BP*: since different health professionals will be measuring BP in the patients for the duration of the study, a certain degree of inter-observer variability will be unavoidable. To minimise this variability, all health professionals will receive training on the technique to measure BP recommended by the reference clinical practice guidelines. Similarly, health professionals will be offered the possibility to attend training workshops to unify criteria for BP measurement. To further reduce the possibility of overlap between the control and intervention groups, the PHCTs that will participate in the study have been selected from different PHC administrative areas.

In conclusion, the implementation and evaluation of the effectiveness of a quality improvement plan may create a working routine in PHCTs that will improve the degree of control of the hypertensive patients and eventually, improve the health of the population.

## Abbreviations

BP: blood pressure; CR: clinical record; DBP: diastolic blood pressure; PHCT: primary health care team; e-CAP: computerised system of clinical records in the ICS; ICS: Catalan Health Institute; INCOTECA: interventions for control of hypertension in Catalonia; PHC: primary health carel PHCT (EAP): primary health care team; SAP: primary care service; SBP: systolic blood pressure.

## Competing interests

The authors declare that they have no competing interests.

## Authors' contributions

RV is the principal investigator and developed the original idea for the study. The study design was further developed by RV, MR, LM, OC, NA, GP, AF and JP. The following authors have contributed to the design and planning of the intervention described in this study: RV, MR, LM, OC and NA (training of the participant doctors, nurses and administrative staff, support material, etc). GP developed the analytical plan. RV, MR, LM, GP, AF, JP and JB will carry out the analysis. All authors have read, corrected draft versions and approved the final version.

## Pre-publication history

The pre-publication history for this paper can be accessed here:


